# TA-AgNPs/Alginate Hydrogel and Its Potential Application as a Promising Antibiofilm Material against Polymicrobial Wound Biofilms Using a Unique Biofilm Flow Model

**DOI:** 10.3390/microorganisms10112279

**Published:** 2022-11-16

**Authors:** Oranee Srichaiyapol, Sarah E. Maddocks, Saengrawee Thammawithan, Sakda Daduang, Sompong Klaynongsruang, Rina Patramanon

**Affiliations:** 1 Department of Biochemistry, Faculty of Science, Khon Kaen University, Khon Kaen 40002, Thailand; 2 Microbiology and Infection Research Group, Cardiff School of Health Sciences, Cardiff Metropolitan University, Cardiff CF5 2YB, UK; 3 Faculty of Pharmaceutical Sciences, Khon Kaen University, Khon Kaen 40002, Thailand; 4 Protein and Proteomics Research Center for Commercial and Industrial Purposes (ProCCI), Khon Kaen University, Khon Kaen 40002, Thailand; 5 Program Management Unit for Human Resources and Institutional Development, Research and Innovation (PMU-B), Bangkok 10330, Thailand

**Keywords:** hydrogel, silver nanoparticles, antibacterial activity, biofilm eradication, wound care

## Abstract

The presence of biofilm within a chronic wound may delay the healing process. Thus, control of biofilm formation and providing bactericidal effect are crucial factors for wound healing management. Alginate-based nanocomposite hydrogels have been suggested as dressing materials for wound treatment, which are employed as a biocompatible matrix. Therefore, in this study, we aimed to develop a biocompatible antimicrobial wound dressing containing AgNPs and demonstrate its efficacy against polymicrobial wound biofilms by using a biofilm flow device to simulate a chronic infected, exuding wound and specific wound environment. The results from agar well diffusion, the Minimum Inhibitory Concentration (MIC) and Minimum Bactericidal Concentration (MBC) assays showed that TA-AgNPs exhibited antibacterial activity against wound pathogens. Additionally, the Minimum Biofilm Eradication Concentration assay (MBEC) demonstrated it could impair biofilm formation. Importantly, our TA-AgNPs/Alginate hydrogel clearly showed antibacterial activities against *Streptococcus pyogenes*, *Staphylococcus aureus* and *Pseudomonas aeruginosa*. Furthermore, we used the biofilm flow device to test the topical antimicrobial hydrogel against a three-species biofilm. We found that TA-AgNPs/Alginate hydrogel significantly showed a 3–4 log reduction in bacterial numbers when applied with multiple doses at 24 h intervals, and was especially effective against the chronic wound pathogen *P. aeruginosa*. This work highlighted that the TA-AgNPs/Alginate hydrogel is a promising material for treating complex wound biofilms.

## 1. Introduction

Chronic wounds are of worldwide significance and their management is incredibly costly. Retrospective analysis in 2018 of Medicare beneficiaries identified that around 8.2 million people had wounds with or without infections. Medicare cost estimates for acute and chronic wound treatments ranged from $28.1 billion to $96.8 billion. The highest expenses were for surgical wounds, followed by diabetic foot ulcers, with a higher trend toward costs associated with outpatient wound care compared with inpatient [1]. Most chronic wounds do not heal due to a secondary infection, which impairs the repair process. Moreover, most of these wounds are infected with bacteria that are resistant to commonly used antibiotics. The WHO estimated that around 500,000 people worldwide are infected with multi-resistant bacteria [2]. The entry of microorganisms into the wound can cause harmless wound contamination, chronic or persistent infection, or in severe cases, serious systemic disease. The colonizing bacteria grow within a biofilm state to establish a barrier to healing by promoting a continuous inflammatory state in the wound, resulting in damage to the localized tissue [3]. Hence, better properties of wound dressing have been intensively developed to prevent a wound shifting from a contaminated to an infected state. Recently, the most promising wound dressings have been modified with biocompatible biopolymers and low toxic nanomaterials for improving properties, which could enhance the wound healing process [4].

Alginate is a linear anionic biopolymer, and its beneficial physical and biological properties are useful in various biomedical applications. This biopolymer consists of α-L-guluronic acid and β-D-mannuronic acid units in different proportions and successive arrangements. Due to the capacity of alginate for bioresorption of the constituent materials, it has been widely used for wound care products. Furthermore, the advantageous properties of alginate as a wound dressing are biocompatibility, nontoxicity, biodegradability, immunogenicity, good film forming and hemostatic potential [5]. Wound dressings should ideally comprise of impermeability to water and bacteria, allowing for gaseous exchange, absorption and retention of exudate, removal of toxic substances, pain relief and comfort, as well as prevention of trauma on removal. The aim of an alginate dressing in wound care could be useful in the red period of the wound healing process to absorb exudate, keep a moist wound environment and accelerate granulation [6]. While water-insoluble calcium alginate is in contact with wound exudates, calcium ions are released because of the replacement of calcium ions with the sodium ions in body fluids that can act as a hemostatic agent. Sodium alginate fiber then adsorbs exudates and turns itself into a gel, which keeps the moist interface on the surface of the wound [7]. Another advantage of ideal wound dressing is a good bacterial barrier against microbe penetration. [8].

Metal nanoparticles have been the most studied in relation to antimicrobial potency in wound healing. Among them, silver nanoparticles (AgNPs) have been used as antibacterial agents for wound and burn dressings [5]. Indeed, numerous studies have investigated that the physicochemical features that affect the antibacterial efficacy of AgNPs include shape, size, surface charge, concentration, the release of Ag^+^ ion, the purification before use and their colloidal state [9,10,11]. These NPs have been reported to have efficacy against planktonic bacteria both in vitro and in vivo environments, and have been shown to be very effective in reducing biofilms on medical devices [6]. Many researchers are looking for a promising antibacterial agent with low toxicity towards humans against multidrug-resistant bacteria (MDR) [12,13]. The efficiency of AgNPs in inhibiting the production of biofilm has been widely evaluated with multidrug-resistant bacterial biofilms, such as antibiofilm and antivirulence potential against MDR *Acinetobacter baumannii*, as well as some studies on the effect of AgNPs on biofilm formation and EPS production of MRD *Klebsiella pneumoniae* [14,15]. Inhibition of bacterial adhesion by these NPs is a key mechanism that enables them to prevent biofilm formation. In comparison to antibiotics, AgNPs may infiltrate into the matrix, destroy the extracellular polymer substance (EPS) and eventually destroy the bacteria within the biofilm [8,16]. In a previous study, AgNPs were approved to reduce the biofilm biomass within 24 h through their smaller size and penetrating ability inside the established biofilm. AgNPs in the sizes of 1 to 100 nanometers can inhibit the biofilm production of *P. aeruginosa* and *S. epidermidis*, as well as which AgNPs, with an average diameter of 25.2 ± 4 nanometers, can effectively inhibit the production of biofilms in *P. aeruginosa* [17]. Another interesting property of AgNPs is being an effective efflux pump inhibitor. To date, previous reports have proved that the use of metal NPs can cause the loss of proton motive force (PMF), which is essential for the normal functioning of many bacterial efflux pumps [18]. The study documented by Mishra et al. [19] found that AgNPs exhibited modulatory effects on the AcrAB-TolC efflux pump in MDR Enterobacter cloacae. Moreover, AgNPs also disrupted the MexAM-OPrM efflux pump kinetics in *P. aeruginosa* by terminating the proton gradient and deteriorating the PMF of the efflux pump system [20]. Crucially, resistance was not induced in *B. pseudomallei* toward AgNPs in the 30th passage, indicating an effective efflux-pump-inhibiting effect against these bacteria after prolonged exposure to AgNPs at sublethal concentrations [17]. However, when AgNPs are developed for human use, their toxicity for mammalian tissues must be considered [21]. Few studies have been reported on the cytotoxicity of AgNPs to cervical cancer cells (HeLa), human lung carcinoma (A549) and human hepatocellular carcinoma (Hep-G2) [22]. Moreover, noncytotoxic AgNPs were demonstrated by Skora and co-workers in 2021 to study the cytotoxicity in four different cell lines, mouse embryonic fibroblasts (NIH 3T3), human keratinocytes (HaCaT), human osteosarcoma (U-2OS) and human non-small cell lung carcinoma (NCI-1299). They found that the number of live cells was still around 95% when exposed to 0.5 mg/mL of AgNPs. Therefore, this finding confirms the previous study and exhibits no or low toxicity in the range of 0.125–0.5 mg/mL of AgNPs [23]. This result is in line with the study reported by Senthil et al., that the green synthesized AgNPs exhibited less cytotoxicity effect on HaCaT cells [24]. 

Recently, several in vitro biofilm models have been successfully modified to study wound biofilms. One of these promising systems was fabricated by Duckworth and his co-workers in 2018. The Duckworth Biofilm Device (DBD) is a unique biofilm flow system that is used to grow several biofilms in repeatable separate channels. This device allows for ease of sampling during experiments without disrupting continuing biofilm growth. It can be used to test the application of wound dressings against wound biofilms with single or polymicrobial biofilms [25]. Moreover, a relevant previous study has demonstrated the efficacy of topical antimicrobial treatments using two- and fives-species chronic wound biofilms by using the DBD, showing that hypochlorous acid (HOCl) gel is a promising treatment for polymicrobial wound biofilms, especially effective against *P. aeruginosa* [26].

In this study, we developed antimicrobial hydrogel-containing AgNPs to demonstrate their efficacy against polymicrobial wound biofilms. Therefore, this study aims to evaluate the antimicrobial activity of tannic-acid-stabilized silver nanoparticles (TA-AgNPs) contained in alginate hydrogel on killing wound biofilms by using the Duckworth biofilm flow device.

## 2. Materials and Methods

### 2.1. Preparation of Culture Media and Strains of Bacteria

Three strains of wound pathogens were used in this study, *Streptococcus pyogenes* MGA S6180 (originally isolated from a wound), *Staphylococcus aureus* EMRSA-15 and *Pseudomonas aeruginosa* ATCC 9027 (type strains originally of skin origin). All strains were cultured at 37 °C in Mueller Hinton Broth (MHB, HiMedia Laboratories Pvt. Ltd., Bengaluru, India). For the polymicrobial biofilms, all bacterial species were cultured at 37 °C in Tryptic Soya Broth (TSB, Oxoid, UK). Testing with a biofilm flow device, mixed-species biofilms were cultured and set in collagen-agar matrices (1% agar containing 50 µg/mL of collagen solution from bovine skin) to promote growing static biofilms. Each bacterial strain was isolated for total viable counts on the following selective agar: *S. pyogenes* on Streptococcus Selective Agar (Fluka Analytical), *S. aureus* on Baird Parker Agar (Millipore, Germany) and *P. aeruginosa* on Cetrimide Agar (Millipore, India).

### 2.2. Preparation of Antimicrobial Agents

Polymyxin B sulfate salt (Sigma Aldrich, Singapore) and Ciprofloxacin Hydrochloride (MP Biomedicals, LLC, France) were prepared in sterile deionized water in the range of a final concentration of 4–1024 µg/mL. Tannic acid powder and tannic-acid-stabilized AgNPs were provided by Prime Nanotechnology Co., Ltd. (Bangkok, Thailand), our collaborator, with a stock concentration of 10,000 mg/L. AgNPs solution for use was the same preparation as the method above. 

### 2.3. Characterization of TA-AgNPs

The same stock of TA-AgNPs as studied in our previous work [17] was used. The 16 µg/mL of TA-AgNPs suspension was diluted in sterile deionized water. The plasmon extinction spectra of AgNPs were measured by UV-Vis spectrophotometer (SPECTROstar Nano, BMG Labtech, Ortenberg, Germany). Other physiochemical-characterized techniques of TA-AgNPs have been performed and described in our previous work [17].

### 2.4. Agar Well Diffusion Assessment 

The antimicrobial activity of TA-AgNPs was tested by following the agar well diffusion method [27,28]. A fresh of 100 µL of bacterial inoculum was spread over the entire agar media surface. A hole of 6 mm in diameter was aseptically punched with a sterile tip and a volume of 30 µL of TA-AgNPs at a final concentration of 128 and 256 µg/mL was added into the wells. The positive control wells for Gram-positive and Gram-negative bacteria were loaded with 8 µg/mL of Ciprofloxacin and 64 µg/mL of Polymyxin B, respectively. The 256 µg/mL of tannic acid and sterile deionized water were used as a negative control. The plates were incubated overnight at 37 °C and the zone of inhibition (ZOI; mm) was then measured and recorded. 

### 2.5. Minimum Inhibitory Concentration (MIC) and Minimum Bactericidal Concentration (MBC) Determination by Broth Microdilution Assay

The MICs and MBCs were performed by broth microdilution method, as recommended by the Clinical and Laboratory Standards Institute [29]. The overnight inoculum was freshly adjusted in MHB to McFarland 0.5 turbidity standard. An equal volume of adjusted bacterial suspension (50 µL) and a range of final concentration from 4 to 256 µg/mL of TA-AgNPs (50 µL) were added to each well of the 96-well plate. The plates were incubated overnight at 37 °C. Ciprofloxacin and Polymyxin B were used as a positive control for Gram-positive and Gram-negative bacteria, respectively. For MBCs, the method of Sengyee et al. [30] was slightly modified. The overnight treated bacteria with no turbidity in the well (10 µL) were dropped on Mueller Hinton Agar (MHA). The Petri dishes were then incubated overnight at 37 °C. The MIC endpoint is the lowest concentration of agent where no visible growth is seen in the microtiter plate. When 99.9% of the bacterial population is killed at the lowest concentration of an antimicrobial agent, it is termed as MBC endpoint [31].

### 2.6. Minimum Biofilm Eradication Concentration (MBEC) Assay

Minimum biofilm eradication concentration (MBEC) is defined as the lowest concentration of an antimicrobial agent required to eradicate biofilm [32]. To promote the biofilm formation of Gram-positive bacteria (*S. pyogenes* and *S. aureus*), we performed a collagen coating method as described by Birkenhauer et al. [33]. The 96-well plates were coated with 50 µg/mL of collagen solution from bovine skin (Sigma-Aldrich, St. Louis, MO, USA). A total of 200 µL of collagen solution was added to each well, then the plate was covered and incubated overnight at 4 °C. After incubation, the coating solutions were discarded, and the wells were then rinsed twice with sterile deionized water. The microdilution assay was performed to test the biofilm eradication activity of TA-AgNPs against *S. pyogenes*, *S. aureus* and *P. aeruginosa*, as modified from Wu et al. and Sabino et al. [34,35]. The overnight cultures were freshly seeded in tryptic soya broth (TSB; Oxoid, England, UK) supplemented with 1% glucose (Sigma-Aldrich, St. Louis, MO, USA) at a cell density of 1 × 10^6^ CFU/mL. An equal volume of adjusted bacterial suspension (50 µL) and a range of final concentrations from 4 to 256 µg/mL of TA-AgNPs (50 µL) were added to each well of the uncoated 96-well plate *(P. aeruginosa)* and the coated plates with collagen (*S. pyogenes* and *S. aureus*). The plates were incubated at 37 °C for 24 h. Then, the wells were washed three times with sterile deionized water to remove non-adherent cells and then stained with 0.1% crystal violet (Sigma-Aldrich, St. Louis, MO, USA) for 5 min. The wells were washed three times with phosphate buffer saline (Sigma-Aldrich, St. Louis, MO, USA) and destained with 7% (*v*/*v*) acetic acid. The plates were read using a 595 nm optical density (OD) spectrophotometer (SPECTROstar Nano, BMG Labtech, Ortenberg, Germany). The negative control was TSB supplemented with 1% glucose. The MBEC was defined as the lowest concentration of antimicrobial agents that led to a final OD similar to the negative control (TSB with 1% glucose) [35].

### 2.7. Preparation of TA-AgNPs-Containing Alginate Gels

The aqueous suspension of tannic-acid-stabilized silver nanoparticles (TA-AgNPs) from a stock solution was prepared to reach a final concentration of 500 µg/mL. A stock solution (50 mL) of 0.1 M CaCl_2_ was prepared in sterile DI water and aseptically filtrated with a 0.02-micron syringe filter before use. The AgNPs/Alginate hydrogel was slightly modified according to the preparation method described by Porter et al. [36]. Sodium alginate powder (Protanal, FMC Biopolymer, Ayrshire, Scotland, UK) was hydrated by adding 30 mL (1% *w*/*v*) of TA-AgNPs suspension while continuously stirred on a stirrer. The mixture was stirred for 30 min or until the alginate was homogeneously dissolved. Then, a small volume (0–5 mL) of 0.1 M CaCl_2_ (5 mL in total) was added drop-wise to the mixture while being automatically stirred on a magnetic stirrer. The hydrogel immediately formed a dark brown gel colored from TA-AgNPs. The TA-AgNPs/Alginate hydrogel was then kept in a sterile vial protecting it from light at room temperature until use.

### 2.8. Antibacterial Activity Test of Antimicrobial Hydrogels

To evaluate the antibacterial activity of TA-AgNPs/Alginate hydrogel, the agar-well diffusion method was performed according to Mekkawy et al. [37]. The overnight cultures were adjusted to 1 × 10^7^ CFU/mL and seeded on MHA media. A hole of 6 mm in diameter was aseptically punched with a sterile tip and the appropriate amount of TA-AgNPs/Alginate hydrogel was then added to fit in the hole. The plates were incubated at 37 °C for 24 h. The diameter of the inhibition zone (mm) was measured to evaluate antibacterial activity. An amount of 1% gel control (blank hydrogel formulation) was tested as a negative control. The positive control used an antibiotic ointment (Neosporin). 

### 2.9. Biofilm Cells Inhibiting test of TA-AgNPs/Alginate Hydrogels Using Duckworth Biofilm Flow Device (DBD)

The setting up and running of the Duckworth biofilm flow device was prepared following the method by Duckworth et al. [25]. The autoclavable 3D printed DBD and tubing were connected to a bottle of fresh TSB supplemented with 1% glucose (pumping in) and spent media was pumped out by a peristaltic pump (Watson Marlow Pump, Falmouth, UK) with a flow rate of 0.332 mL/min (equivalent of 0.083 mL/min through each channel) to a waste bottle. Twelve disks of 1.5% (*w*/*v*) noble agar were aseptically cut using a sterile 8 mm biopsy punch and transferred to the device using sterile forceps. To prepare the bacteria-collagen agar matrices, a mixed bacterial suspension (1:1 ratio) was equilibrated to a final concentration of 1 × 10^6^ CFU/mL in a pre-warmed TSA media supplemented with 1% glucose and 50 µg/mL of collagen from bovine serum. The bacteria-collagen agar mixture was poured into a 24-well plate and solidified at room temperature, then cut with a sterile 8 mm biopsy punch. A bacteria-collagen agar matrix was placed on top of each noble agar in a separate channel of the DBD. To test for biofilm killing, 0.25 g of TA-AgNPs/Alginate gel was added to each treated biofilm at a time point of 5 h, 24 h and 48 h. Established biofilms with no treatment were used as untreated control. At 24, 48 and 72 h, the bacteria-collagen agar was transferred from the device to 1 mL of sterile PBS to homogenize the biofilm and was enumerated by TVC. For sterilization at the end of each experiment, the tubing and device were decontaminated in 10% Gerrard Ampholytic Surface Active Biocide (GASAB) disinfectant for 24 h before autoclaving.

### 2.10. Biofilm Recovery Assessment by Total Viable Counts (TVCs)

The assessment of biofilm cells recovery was examined by the method described by Nedelea et al. [26]. Bacteria-collagen agar matrices were collected at 24, 48 and 72 h. Then, each agar matrix was placed and homogenized by a sterile glass rod in a homogenizer glass tube containing 1 mL of sterile phosphate-buffered saline. Serial dilutions were performed in sterile DI water from 10^−1^ to 10^−8^. The diluted bacterial suspension (5 µL) was then dropped on each selective media for each bacterial strain. The plates were incubated at 37 °C for 24 h. The cultured biofilms for 24, 48 and 72 h, without adding any agent, were used as the untreated control. 

### 2.11. Statistical Analysis

The data were analyzed using at least two independent experiments in triplicate and presented as the mean ± SD. A one-way analysis of variance (ANOVA) was used to determine a significance test between groups using the Statistical Package for the Social Science (SPSS) version 28.0 (IBM SPSS Statistics, Armonk, NY, USA).

## 3. Results

### 3.1. Characterization of TA-AgNPs

**Figure 1:**
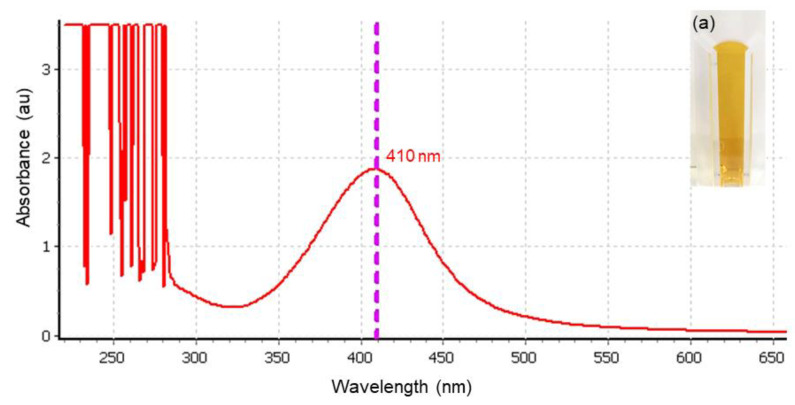
UV-Vis spectra of silver nanoparticles at 410 nm; inset (**a**): TA-AgNPs colloid showed a yellow color.

Tannic-acid-stabilized silver nanoparticles (TA-AgNPs) were provided by our collaborator, Prime Nanotechnology Co, Ltd. (Bangkok, Thailand). A concentration of TA-AgNPs at 16 µg/mL was used as an optimal concentration throughout characterization studies. The color of AgNPs colloids was yellow, as shown in Figure 1a (inset). The UV-Vis spectrum showed a single peak of maximum absorption at 410 nm, corresponding to the surface plasmon resonance (SPR band) of AgNPs at 37 °C (Figure 1). For other physiochemical characterizations of TA-AgNPs, we performed and described these in our previous study [17]. In brief, as shown and additionally described in Figure S2, TA-AgNPs had a spherical shape with an average size of 7.99 nm, and were well monodispersed. They had average hydrodynamic diameters of 101 nm and high particle homogeneity. TA-AgNPs had a negative charge from tannic acid contribution, electrostatically stabilized on AgNPs surfaces, and revealed stability due to their zeta potential of −47.63 mV. 

### 3.2. TA-AgNPs Exhibited Antibacterial Activity against Bacteria Causing Wound Infection

Agar well diffusion was performed to evaluate a zone of inhibition. *S. pyogenes* and *S. aureus* represented Gram-positive bacteria, and *P. aeruginosa* represented Gram-negative bacteria. The inhibition zones of TA-AgNPs against three bacteria were shown at concentrations of both 128 µg/mL and 256 µg/mL, while 256 µg/mL of tannic acid showed no inhibition zone against these three bacteria. These results could indicate that the main antibacterial effect is exhibited by AgNPs. Furthermore, sterile distilled water was used as a negative control and showed no inhibition zone, while 8 µg/mL of ciprofloxacin and 64 µg/mL of polymyxin B were used as a positive control for Gram-positive and Gram-negative bacteria, which clearly showed an inhibition zone, respectively (Table 1 and Figure S1).

### 3.3. Minimum Inhibitory Concentration (MIC) and Minimum Bactericidal Concentration (MBC) Values of TA-AgNPs against Wound Bacteria

For the minimum inhibitory concentration (MIC) and the minimum bactericidal concentration (MBC) of TA-AgNPs against *S. pyogenes, S. aureus* and *P. aeruginosa*, the broth microdilution method was performed. The results showed that TA-AgNPs could inhibit the growth of *S. pyogenes* and *S. aureus* at the MIC of 4 and 32 µg/mL and the MBC of 8 and 64 µg/mL, respectively. While the MIC and MBC of TA-AgNPs against *P. aeruginosa* were 64 and 128 µg/mL, respectively (Table 2). Ciprofloxacin and polymyxin B were used as a positive control for Gram-positive and Gram-negative bacteria, respectively. 

### 3.4. TA-AgNPs Could Reduce Biofilm Formation and Inhibit the Planktonic Bacterial Growth

**Figure 2:**
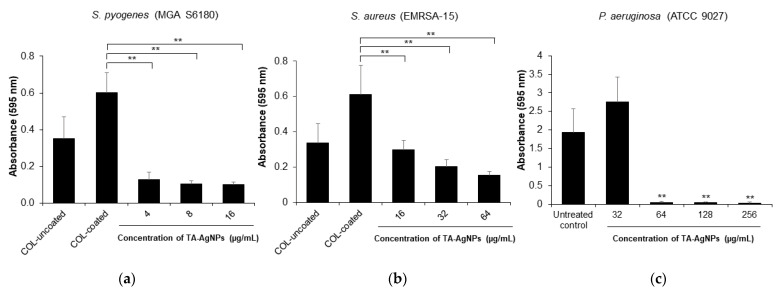

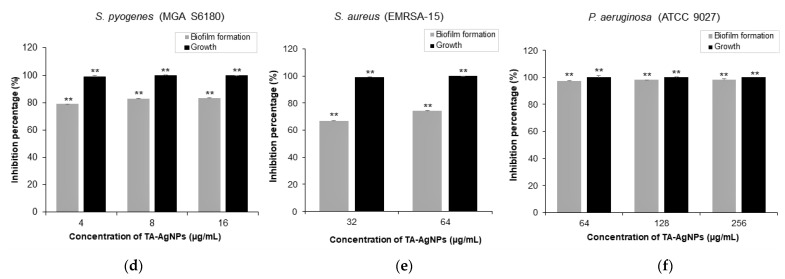
Effect of TA-AgNPs on the biofilm development at 595 nm: *S. pyogenes* (**a**); *S. aureus* (**b**); and *P. aeruginosa* (**c**). Inhibition effect of TA-AgNPs on the bacterial growth and biofilm formation of *S. pyogenes* (**d**); *S. aureus* (**e**); and *P. aeruginosa* (**f**). COL: collagen. ** *p* < 0.01, compared with control.

A minimum biofilm eradication concentration (MBEC) is described as the lowest concentration of an antimicrobial agent required to eradicate biofilm [32]. The microdilution assay was performed to test the biofilm eradication activity of TA-AgNPs. MBEC was defined as the lowest concentration of antimicrobial agents that led to a final OD similar to the negative control (broth only) [35]. To promote the biofilm formation of Gram-positive bacteria, the microtiter plates were coated with collagen solution. The results showed that the biofilm formation of *S. pyogenes* and *S. aureus* were increased when they were cultured in collagen-coated plates (Figure 2a,b). In terms of biofilm eradication, the results exhibited that TA-AgNPs significantly (*p* < 0.01) inhibited the growth of planktonic bacteria and eradicated the established biofilm of *P. aeruginosa* with the MBEC value of 128 µg/mL (Table 3, Figure 2c,f), while the MBEC of TA-AgNPs against *S. pyogenes* and *S. aureus* were higher than 16 and 64 µg/mL, respectively (Table 3). As shown in Figure 2a,b, when treated with TA-AgNPs ranging from 4 to 16 µg/mL and 32 to 64 µg/mL, the biofilm biomass of *S. pyogenes* and *S. aureus* were significantly reduced, respectively (*p* < 0.01). In Figure 2d, the biofilm formation of *S. pyogenes* was significantly inhibited in a range from 78.97% to 83.45% when TA-AgNPs increased from 4 to 16 µg/mL (*p* < 0.01). For *S. aureus*, as shown in Figure 2e, the biofilm formation was significantly inhibited to 66.84% and 74.51% when treated with TA-AgNPs at the concentration of 32 and 64 µg/mL, respectively (*p* < 0.01). To determine the effect of TA-AgNPs on growth inhibition, all tested concentrations of TA-AgNPs (as shown in Figure 2d–f) could significantly inhibit the growth of all three planktonic bacteria in the range of 98.93% to 100% (*p* < 0.01). These results demonstrated that TA-AgNPs could reduce the biofilm biomass and affect the bacterial growth.

### 3.5. Antibacterial Activity of Antimicrobial TA-AgNPs/Alginate Hydrogels

**Figure 3:**
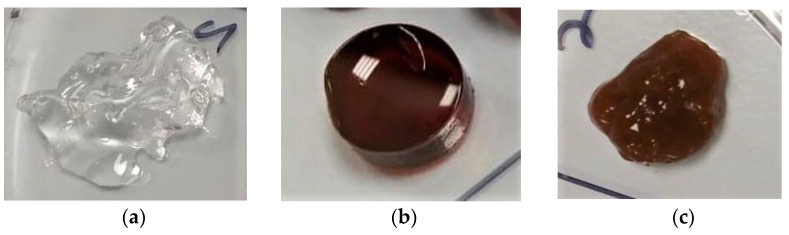
Photographs of the hydrogels: 1% alginate hydrogel (**a**); TA-AgNPs gel (**b**); and TA-AgNPs-containing alginate hydrogel (**c**).

**Figure 4:**
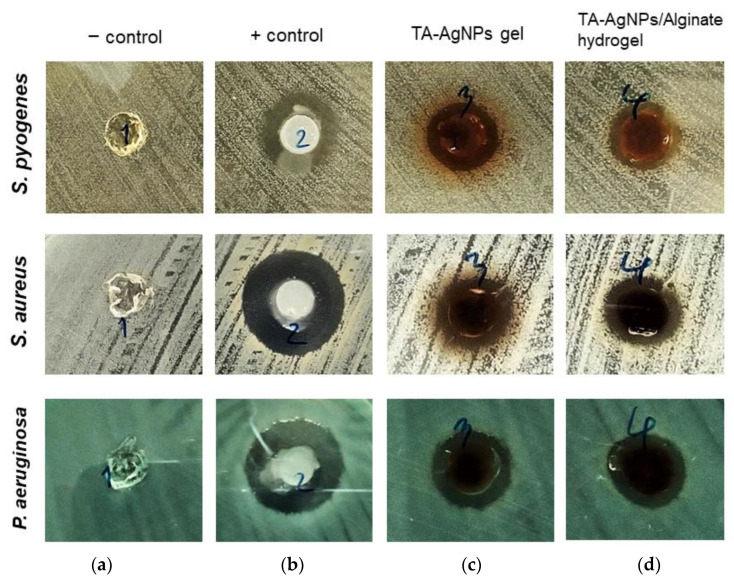
Diameter of zone of inhibition (mm): *S. pyogenes* (top); *S. aureus* (middle); and *P. aeruginosa* (bottom), inset: No.1: 1% alginate gel control (negative control) (**a**); No.2: antibiotic ointment (positive control, Neosporin^®^) (**b**); No.3: 512 µg/mL of TA-AgNPs gel (**c**); and No.4: 512 µg/mL of TA-AgNPs/Alginate hydrogel (**d**).

The characteristics of TA-AgNPs/Alginate hydrogel are shown in Figure 3. The 1% alginate hydrogel without TA-AgNPs was colorless (Figure 3a), the gel containing TA-AgNPs was dark brown (Figure 2b) and the alginate hydrogel containing TA-AgNPs formed a dark brown turbid gel (Figure 3c). The agar well diffusion method was used to determine the antibacterial activity of TA-AgNPs/Alginate hydrogel against three bacterial strains. The alginate hydrogel without TA-AgNPs was used as a negative control and a positive control was antibiotic ointment (Neosporin^®^). As shown in Figure 4a and Table 4, the alginate hydrogel did not show any antibacterial activity. While the gel containing TA-AgNPs and the TA-AgNPs/Alginate hydrogel clearly showed antibacterial activities (Figure 4c,d). The inhibition zone of TA-AgNPs/Alginate hydrogels were 9.75 ± 0.27 mm, 11.21 ± 0.70 mm and 10.75 ± 0.88 mm against *S. pyogenes*, *S. aureus* and *P. aeruginosa*, respectively (Table 4).

### 3.6. Polymicrobial Biofilms Development in the DBD Model

**Figure 5:**
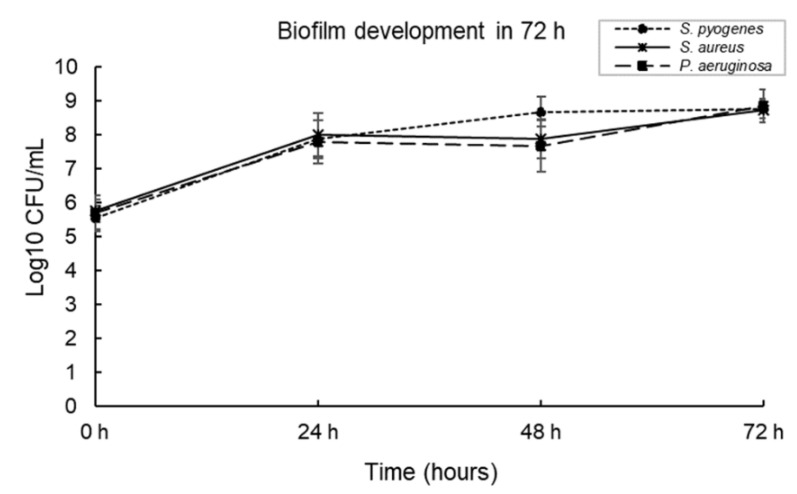
A three-species biofilm grown in the Duckworth Biofilm Device (DBD) for 72 h at 33 °C.

Three-species biofilms were cultured in the DBD model at 33 °C for 72 h and enumerated at 24-h intervals. *S. pyogenes*, *S. aureus* and *P. aeruginosa* were used as representative wound pathogens. As shown in Figure 5, a three-species biofilm could grow in this model in a steady state. All three bacteria were detectable at the 24 h, 48 h and 72 h time points. 

### 3.7. Effectiveness of TA-AgNPs/Alginate Hydrogel against a Three-Species Biofilm

**Figure 6:**
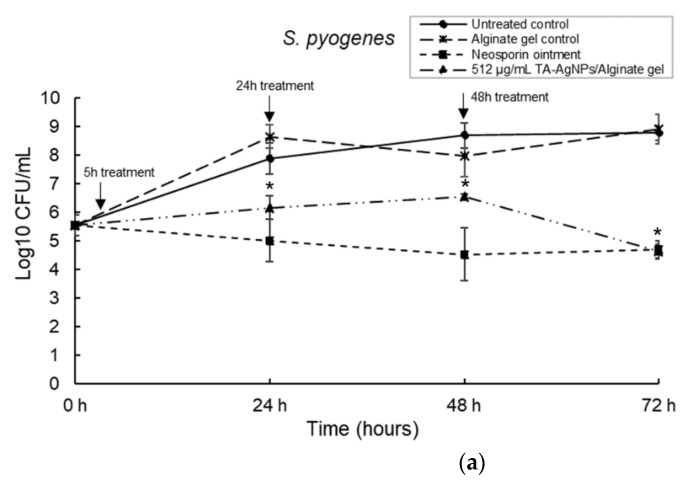

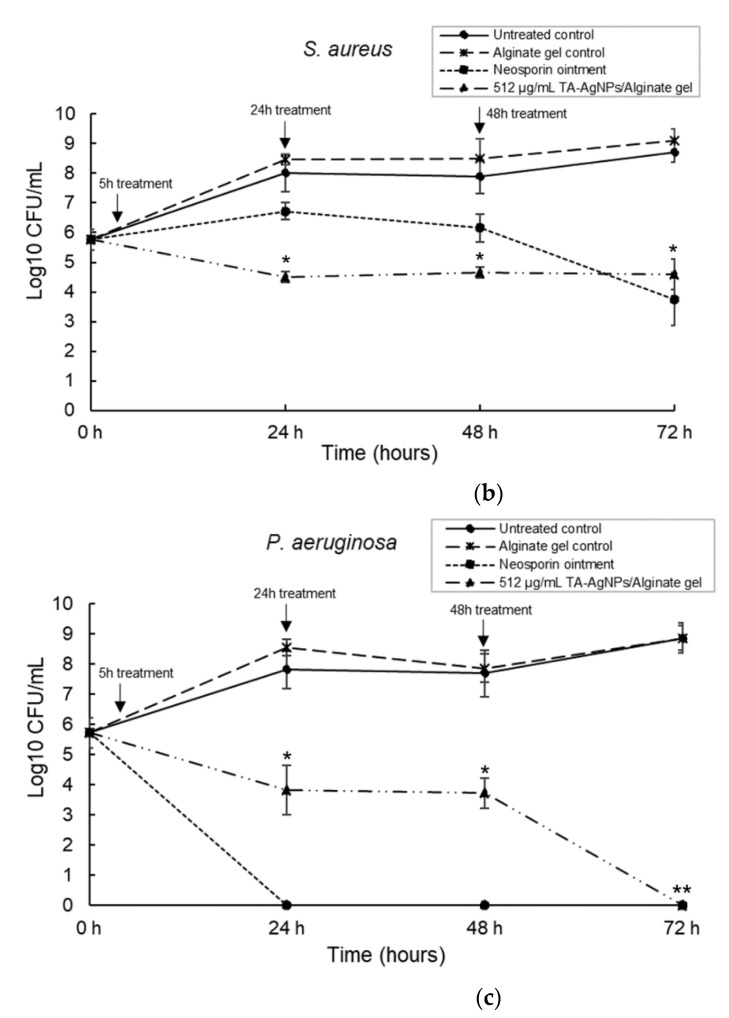
Multiple doses of TA-AgNPs/Alginate hydrogels treated at 5, 24 and 48 h (indicated by arrows) against a three-species biofilm: *S. pyogenes* (**a**); *S. aureus* (**b**); and *P. aeruginosa* (**c**).

The first dose of antibacterial alginate hydrogel was applied at 5 h; this time point was taken to be the pre-Gram negative shift and allowed for the establishment of the biofilm [26]. The untreated biofilm was used as a control to compare any growth changes. Three-species biofilms comprised of *S. pyogenes*, *S. aureus* and *P. aeruginosa* and were cultured in the DBD model for 5 h, 24 h and 48 h prior to treatment with three separate doses of TA-AgNPs/Alginate hydrogel. Antibiotic ointment (Neosporin^®^) was used as a positive control and the alginate hydrogel without TA-AgNPs was used as a negative control. To count the recovery cells, TVC was performed at 24, 48 and 72 h. The application of TA-AgNPs/Alginate hydrogel against *S. pyogenes* at 5 h and 24 h resulted in significantly (*p* < 0.05) reduced bacterial numbers. At 48 h, the results showed a 3-log reduction in bacterial numbers compared to the untreated control (*p* < 0.05) (Figure 6a). For *S. aureus*, as shown in Figure 6b, the number of bacteria were significantly (*p* < 0.05) reduced to a 3-log reduction when TA-AgNPs/Alginate hydrogels were applied for three separate doses at 5 h, 24 h and 48 h. Interestingly, among the three-species biofilm, *P. aeruginosa* was no longer detectable after being treated with three doses of TA-AgNPs/Alginate hydrogel and no bacteria recovered in number over 72 h (*p* < 0.01) (Figure 6c). Additionally, no Gram-negative shift was observed when treated with multiple doses of TA-AgNPs/Alginate hydrogel. 

## 4. Discussion

Recently, biofilm has been implicated in most non-healing wounds and wound infections believed to be present in approximately 78% of chronic wounds [38]. The presence of biofilm within a chronic wound may delay the healing process and contribute to keeping the wound in a state of prolonged inflammation by the stimulation of nitric oxide, inflammatory cytokines and free radicals [39,40]. Thus, the control of biofilm formation and bactericidal activity are crucial factors for wound healing management. The most common bacterial pathogens found in the infected wounds are *Pseudomonas aeruginosa*, *Escherichia coli*, *Proteus mirabilis*, *Acinetobacter baumannii/haemolyticus*, *Staphylococcus aureus*, *Streptococcus pyogenes* and *Corynebacterium spp*. [41]. Among these pathogens, *P. aeruginosa* and *S. aureus* are the most commonly co-isolated from polymicrobial infections and have high resistance to several antibiotics, in part due to their biofilm formation [42]. In the present work, we have studied a three-species biofilm comprised of *P. aeruginosa, S. aureus* and *S. pyogenes* because these three bacteria could recover after 24-h topical treatment, as shown in a previous study described by Nedelea et al., indicating their recalcitrance. However, multiple doses of antimicrobial treatment prevented the bacterial recovery, as well as the bacteria not becoming tolerant to the treatment. Thus, these results were used to inform this study [26].

The antimicrobial efficacy of silver nanoparticles (AgNPs) for the treatment of infected wounds has been reported in several studies [43,44,45]. In this study, we received the tannic-acid-stabilized silver nanoparticles (TA-AgNPs) from our collaborative company (Prime Nanotechnology, Thailand, co. Ltd.). These TA-AgNPs showed a maximum absorption peak at 410 nm measured by UV-Visible spectrophotometry (Figure 1), which was a similar pattern to that reported by Liaqat et al. [46]. In our previous work, we found that TA-AgNPs had a spherical shape, were well monodispersed, and had high particle homogeneity and high stability [17]. The clear inhibition zone from the agar well diffusion assay, together with the effective MICs and MBCs values, revealed the biocidal activity of TA-AgNPs against both tested Gram-positive (*S. pyogenes* and *S. aureus*) and Gram-negative bacteria (*P. aeruginosa*). A simple structure model of tannic-acid-stabilized AgNPs is shown in Figure S3. The hydrophobic moieties and hydrophilic shell of tannic acid stabilized on AgNPs surface play an important role in its interaction with lipid and surface proteins in bacterial cells. These polyphenolic features of tannic acid could promote close contact between AgNPs and the bacterial cell surfaces [47,48]. From the MBEC determination, we found that TA-AgNPs could impair biofilm formation of *S. pyogenes*, *S. aureus* and *P. aeruginosa*. Similar to the study of Singh et al. [49], silver nanoparticles have been shown to effectively kill bacterial biofilms against *P. aeruginosa, S. aureus* and other Gram-negative pathogens. Additionally, the efficacy of synthesized AgNPs has been reported to inhibit both planktonic growth and biofilm formation of *P. aeruginosa* [50]. 

Many wound dressings have been developed for protecting a wound from infection and for promoting the wound healing process [51]. Interestingly, AgNPs-based products have been commercialized in the market for wound dressing applications [43]. Silver alginate wound dressings are known to have benefits in wound care with their bioavailability [3]. Hydrogels, such as alginate, are a beneficial type of carrier for antimicrobial agents due to their low price and biocompatibility, as well as their protective and non-toxic matrix for use in biomedical applications [36]. In the present work, we successfully prepared a TA-AgNPs/Alginate hydrogel; the gel formation properly occurred and appeared dark-brown in color. Similar to previous studies, Porter et al. [36] reported that the developed thioctic-acid-capped AgNPs in an alginate hydrogel were made possible by the classic egg-box model crosslinking of calcium alginate. An oxygen atom with L-guluronate chains of alginate can easily form polymer hydrogels through replacement of the sodium cation with divalent or multivalent cations, which act as crosslinkers [52]. Alginate hydrogels crosslinked with divalent calcium cation (Ca^2+^) present a structure in which Ca^2+^ fits into the guluronate block structure like eggs, forming the metal chelation-binding chain that has been termed as the “egg box” model [53]. The TA-AgNPs/Alginate hydrogel have effective antibacterial properties against *S. pyogenes*, *S. aureus* and *P. aeruginosa*. The antibacterial efficacy results in our study corroborated with a study by Diniz et al. [54], in which hydrogels incorporating AgNPs showed inhibitory effects on bacterial growth. Additionally, Porter et al. [36] established that the AgNPs alginate gel exhibited significant cell killing on biofilms of Gram-positive and Gram-negative bacteria. Moreover, the developed TA-AgNPs/Alginate hydrogel in this study was still stable for four months, and exhibited the antibacterial efficacy against the mixed three-species biofilms. Similar studies were also reported by Diniz et al. [54], who developed silver nanoparticles-composing Alginate/Gelatin hydrogel to improve wound healing. They demonstrated that sodium alginate can enhance the stability of AgNPs by directly acting as a stabilizer, as well as avoiding AgNPs aggregation. Moreover, it could help prevent hydrogel degradation from oxidation or exposure to light.

Polymicrobial biofilms are known to be more inherently resistant to antimicrobial treatments than mono-species biofilms. Therefore, we investigated whether the TA-AgNPs/Alginate hydrogel could inhibit the biofilm cells in mixed-species biofilms cultured in a biofilm flow model. A Duckworth device was used to represent a chronic infected, exuding wound in a physiologically relevant wound environment. Using a more realistic chronic wound model provides the same kind of challenges to treatment that real-world wounds present. The reactor was incubated to culture the biofilms at 33 °C to simulate average wound bed temperature [55]. The bacteria immobilized in collagen matrices were continuously cultured with fresh media at a flow rate of 0.332 mL/min in the incubation duration to simulate the flow of an exuding wound [26]. The three-species biofilm in DBD, was maintained in a steady state for 72 h of incubation. This mirrored a previous study [55], which reported that the collagen wound biofilm model facilitates the growth of reproducible biofilms under wound-like conditions. Furthermore, we used the DBD to test the topical antimicrobial hydrogel against the cultured biofilms. The 5-h-established mixed-species biofilms were applied with TA-AgNPs/Alginate hydrogel prior to the Gram-negative shift, to see if treatment could prevent this phenomenon. Gram-negative shift is a well-known phenomenon found in wound infections. Typically, in vitro biofilms cultured for less than 10 h show a predominance of Gram-positive bacteria; after 10 h, Gram-negative bacteria become predominant and remain for the incubation duration [25]. The cultured biofilms at 24 h and 48 h were treated with TA-AgNPs/Alginate hydrogel to determine their efficacy against the established biofilms. Our findings indicate that TA-AgNPs/Alginate hydrogel showed significant reduction in bioburden. In our three-species biofilm model, the results showed a 3–4 log reduction in bacterial numbers when applied with multiple doses of TA-AgNPs/Alginate hydrogel at 5 h, 24 h and 48 h. Interestingly, we found that our developed hydrogel significantly reduced the number of *P. aeruginosa* and did not recover post-treatment. Similar studies have also been reported by Nedelea et al. [26], showing that the topical treatment of HOCl gel using the same DBD model revealed no post-treatment recovery of *P. aeruginosa*. Biofilm destruction by AgNPs was mediated through the binding of AgNPs to the exopolysaccharide matrix and disrupts the biofilm structure by interfering with the peptidoglycan present in bacterial cell walls. Intracellularly, it causes physical damage, ion release, ROS production and leads to DNA damage and oxidative stress [56]. Bacteria can become resistant to these types of antimicrobial stresses, and we suggest that treatment with multiple doses of TA-AgNPs/Alginate hydrogel at 24-h intervals may prevent the bacteria from becoming tolerant to the treatment over time. This work highlighted that the TA-AgNPs/Alginate hydrogel is a promising material for treating complex wound biofilms, and is especially effective against the chronic wound pathogen *P. aeruginosa*. 

## 5. Conclusions

The developed TA-AgNPs/Alginate hydrogel using the classic egg-box model crosslinking of calcium alginate was demonstrated against polymicrobial wound biofilms by using a biofilm flow device. This antimicrobial hydrogel not only significantly reduced the bacterial numbers in established biofilms in a complex chronic wound-biofilm model, but the treatment of the hydrogel at 24-h intervals also helped prevent the bacterial tolerant post-treatment. These findings highlight a desirable feature by which to evaluate the antimicrobial activity of treatments using a kinetics flow device, or studies regarding the development of tolerance in biofilm cultures during treatment of biofilms. Thus, we propose TA-AgNPs/Alginate hydrogel and its potential application as a promising antimicrobial material for wound dressings.

## Figures and Tables

**Table 1:** Diameter of zone of inhibition of TA-AgNPs against bacteria causing wound infection.

Agent (μg/mL)	Diameter of Zone of Inhibition (mm)		
	Gram-Positive Bacteria		Gram-Negative Bacteria
	*S. pyogenes*	*S. aureus*	*P. aeruginosa*
128 μg/mL TA-AgNPs	12.00 ± 0.93	9.25 ± 0.99	8.67 ± 0.52
256 μg/mL TA-AgNPs	12.88 ± 0.64	10.58 ± 0.80	10.25 ± 0.42
8 μg/mL Ciprofloxacin	13.40 ± 1.34	18.33 ± 0.98	n.t. ^1^
64 μg/mL Polymyxin B	n.t.	n.t.	12.92 ± 0.80
256 μg/mL Tannic acid	n.d. ^2^	n.d.	n.d.
Sterile deionized water (DI)	n.d.	n.d.	n.d.

^1^ n.t. = not tested; ^2^ n.d. = not detected.

**Table 2:** MIC and MBC values of TA-AgNPs against bacteria causing wound infection.

Agent (μg/mL)	Gram-Positive Bacteria				Gram-Negative Bacteria	
	*S. pyogenes*		*S. aureus*		*P. aeruginosa*	
	MIC ^1^	MBC ^2^	MIC	MBC	MIC	MBC
TA-AgNPs	4	8	32	64	64	128
Ciprofloxacin	0.25	0.5	<0.25	0.25	n.t. ^3^	n.t.
Polymyxin B	n.t.	n.t.	n.t.	n.t.	4	8

^1^ MIC endpoint is the lowest concentration of agent where no visible growth is seen in the microtiter plate; ^2^ MBC endpoint is when 99.9% of the bacterial population is killed at the lowest concentration of an antimicrobial agent; ^3^ n.t. = not tested.

**Table 3:** MBEC of TA-AgNPs against three wound bacteria.

Agent (μg/mL)	Gram-Positive Bacteria		Gram-Negative Bacteria
	*S. pyogenes*	*S. aureus*	*P. aeruginosa*
TA-AgNPs	>16	>64	128
Ciprofloxacin ^1^	>16	>16	n.t. ^3^
Polymyxin B ^2^	n.t.	n.t.	8

^1^ Ciprofloxacin was used as a positive control for Gram-positive bacteria; ^2^ Polymyxin B was used for Gram-negative bacteria; ^3^ n.t. = not tested.

**Table 4:** Diameter of zone of inhibition of TA-AgNPs gel against bacteria causing wound infection.

Agent (μg/mL)	Diameter of Zone of Inhibition (mm)		
	Gram-Positive Bacteria		Gram-Negative Bacteria
	*S. pyogenes*	*S. aureus*	*P. aeruginosa*
1% alginate gel control	n.d. ^2^	n.d.	n.d.
Antibiotic ointment (Neosporin^®^) ^1^	16.08 ± 0.66	20.07 ± 1.10	19.75 ± 1.44
512 μg/mL TA-AgNPs gel	10.67 ± 0.41	12.36 ± 0.85	13.00 ± 0.71
512 μg/mL TA-AgNPs/Alginate gel	9.75 ± 0.27	11.21 ± 0.70	10.75 ± 0.88

^1^ Antibiotic ointment (Neosporin^®^) was used as a standard antibacterial agent; ^2^ n.d. = not detectable.

## Data Availability

The data presented in this study are available on the request from the corresponding author.

## Supplementary Materials

The following supporting information can be downloaded at: https://www.mdpi.com/article/10.3390/microorganisms10112279/s1, Figure S1: Diameter of zone of inhibition (mm): *S. pyogenes* (a); *S. aureus* (b); and *P. aeruginosa* (c), inset: distilled water (a), 128 µg/mL of TA-AgNPs (b), 256 µg/mL of TA-AgNPs (c), 256 µg/mL of tannic acid (d), 8 µg/mL of ciprofloxacin (e) and 64 µg/mL of polymyxin B (e). Figure S2: Physicochemical characterization of TA-AgNPs: (a) Transmission electron micrograph (TEM) of TA-AgNPs showing a spherical shape and well-monodispersed; (b) average particles size of 7.99 nm; (c) EDX spectrum of AgNPs; (d) the average hydrodynamic diameters of 101 nm showing high particle homogeneity; (e) and a negatively charge from tannic acid contribution produced electrostatically stabilized on AgNPs surfaces and revealed the stability due to their zeta potential of −47.63 mV (Srichaiyapol et al., 2021.). Figure S3: A simple structure model of tannic-acid-stabilized AgNPs (pH = 5.94): the hydrophobic core and hydrophilic shell of tannic acid stabilized on AgNPs surface which are the part that could promote close contact between AgNPs and the bacterial cell surfaces.
