# Supplementary figures and images for: TA-AgNPs/Alginate Hydrogel and Its Potential Application as a Promising Antibiofilm Material against Polymicrobial Wound Biofilms Using a Unique Biofilm Flow Model
Source: Microorganisms. 2022 Nov 16;10(11):2279. doi: 10.3390/microorganisms10112279 (PMC9692730; doi:10.3390/microorganisms10112279)

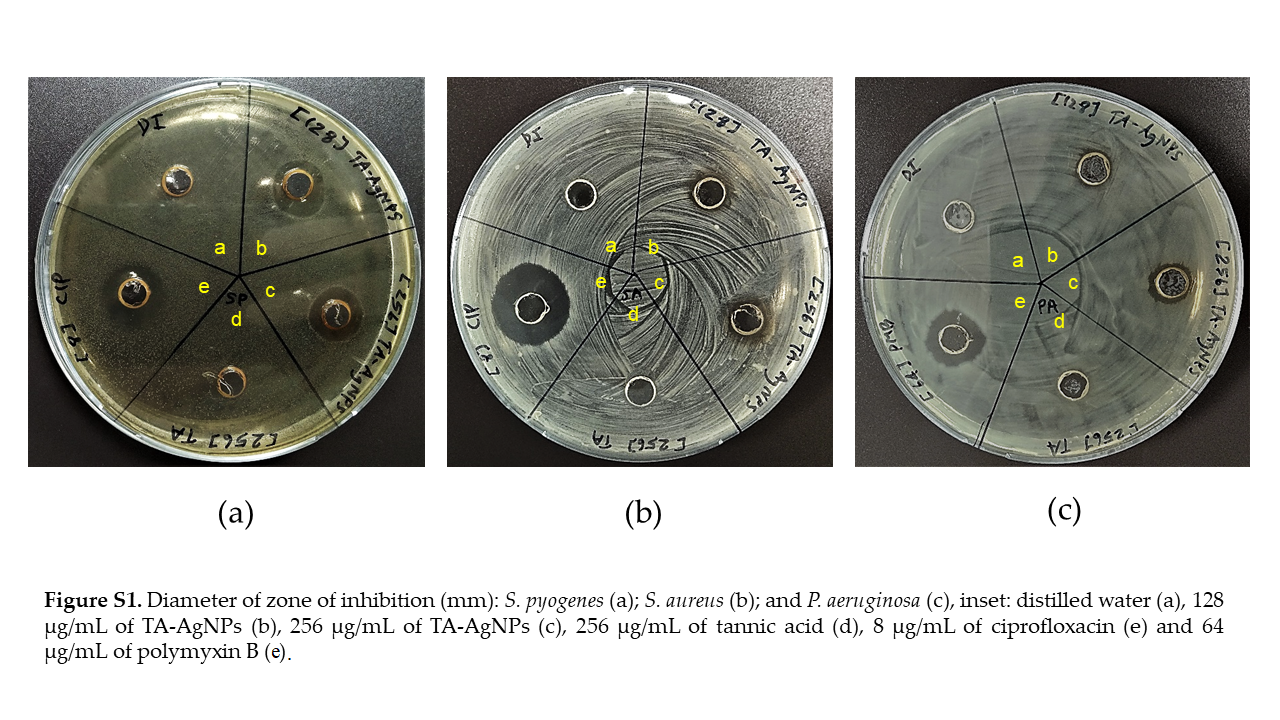

Supplement: Supplementary file 1 [file microorganisms-10-02279-s001.zip › Figure S1.tif]

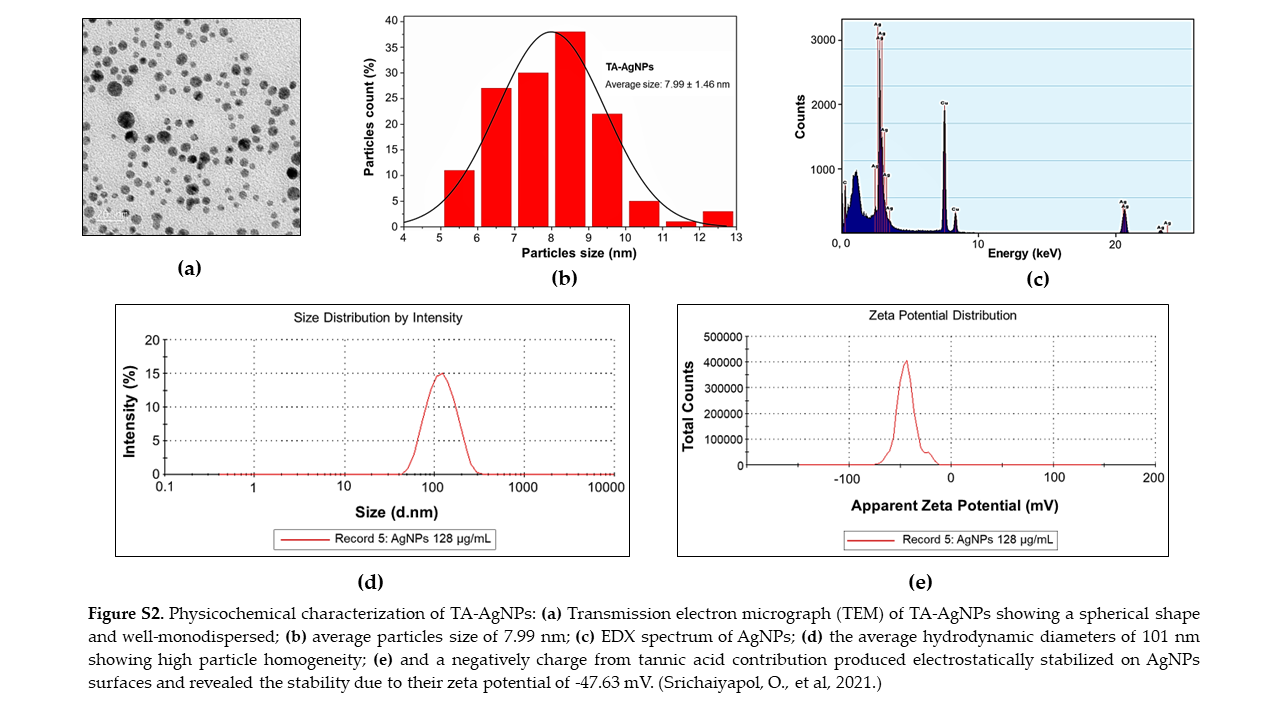

Supplement: Supplementary file 1 [file microorganisms-10-02279-s001.zip › Figure S2.tif]

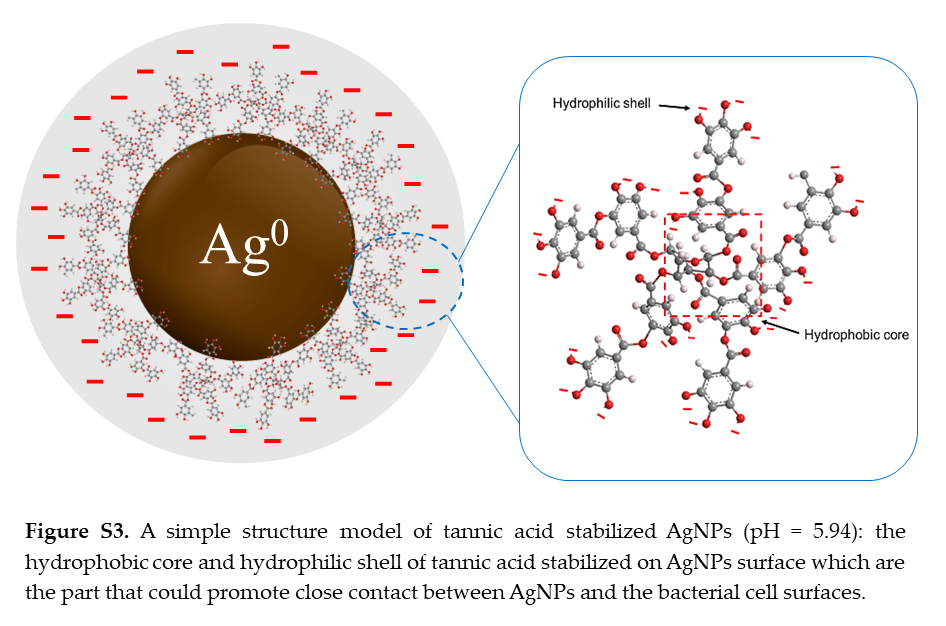

Supplement: Supplementary file 1 [file microorganisms-10-02279-s001.zip › Figure S3.tif]
